# Money, Politics, and Firearm Safety: Physician Political Action Committees in the Era of “This is Our Lane”

**DOI:** 10.1001/jamanetworkopen.2018.7823

**Published:** 2019-02-01

**Authors:** Rebecca M. Cunningham, Marc A. Zimmerman, Patrick M. Carter

**Affiliations:** Department of Health Behavior & Health Education, University of Michigan School of Public Health, Ann Arbor (Cunningham, Zimmerman); Department of Emergency Medicine, University of Michigan School of Medicine, Ann Arbor (Cunningham, Carter).

Firearm injuries are a significant US public health problem, responsible for nearly 40 000 deaths annually.^1^ In 2017, firearms surpassed motor vehicle crashes and are now second only to opioid and other drug-related overdoses as the leading cause of injury-related death.^1^ One of the most appalling aspects of this public health tragedy is the toll that such firearm injuries extract on the most vulnerable populations, particularly children, adolescents, and elderly people. Among children and adolescents, firearms are the second leading cause of death overall and are the leading cause of death for African American youth.^2^ Among elderly people (≥65 years of age), firearms are responsible for 70% of the more than 8200 completed suicide attempts every year.^1^ Apart from the human costs, firearm-related injuries also cost society an estimated $230 billion annually, considering costs for acute medical care, long-term disability and rehabilitation care, lost work and productivity, and criminal justice proceedings.^3^

Health care professionals experience daily the physical and emotional toll of firearm-related injury, whether they are emergency medicine or trauma specialists who care for acutely injured patients in the trauma bay, operating theater, or intensive care units or the internal medicine, pediatric, family medicine, rehabilitation, or psychiatry specialists who serve patients and their families dealing with the aftermath of a firearm injury. The depth of their investment in this issue was recently demonstrated through the nationwide response to the National Rifle Association’s (NRA’s) statement admonishing physicians to “stay in their lane” and avoid commenting on public policies designed to address firearm safety.^4^ Health care professionals responded with social and mass media and journal editorials describing their encounters with patients injured by firearms. Health care professionals demonstrated that contrary to the NRA position, they have an undeniably central role and authority in addressing this public health problem through the direct care that they provide to patients and their families, prevention-based research, and advocacy for policy-level changes that make patients safer.

Although social media have served as a galvanizing force for the health care community, national health care organizations such as the ones in the study reported by Schuur et al^5^ also play an important role to advocate on behalf of their members at the federal and state levels. These organizations have been silent on the issue of firearm violence, often fearful of touching what has become a third rail of medical politics. Driven largely by their memberships’ increasingly vocal concerns and in response to the numerous recent mass shootings, these national membership organizations have begun to take a more outspoken lead in advocatingfor firearm safety. Organizations such as the American Medical Association,^6^ the American College of Emergency Physicians,^7^ and the American College of Surgeons Committee on Trauma^8^ have released new position statements and/or editorials advocating for increased research funding and the introduction of sensible regulatory and enforcement policies at the state and national levels that have demonstrated evidence at curbing firearm violence. The National Academy of Medicine also recently held a conference exploring ways in which health care systems can address the issue of firearm violence at both the individual practitioner and the medical system levels.^9^ Recent events, including the National Institutes of Health-funded research initiatives (eg, Firearm Safety Among Children and Teens Consortium) to build research capacity in this arena, as well as the American Foundation for Firearm Injury Reduction in Medicine (AFFIRM), a coalition of clinicians and researchers across medical specialties committed to reducing firearm violence, are indicators of the beginning of the end of the medical community’s silence on the issue of firearm research and safety.

Although such attention is urgently needed, the article by Schuur et al^5^ highlights the disconnect that currently exists between physician organization political action committee (PAC) priorities and the positions of their membership when it comes to firearm injury prevention. The authors found that even among professional organizations that endorsed the 2015 Firearm-Related Injury and Death in the United States: A Call to Action, there was significant support for candidates with records in opposition to firearm safety policies. As the authors note, this is not a new issue in medicine. For many years, we saw a similar discrepancy between the American Medical Association’s public calls to regulate the tobacco industry and their financial support of politicians who actively voted against such regulations.^10^ This history lesson serves as an important cautionary tale. Schuur et al^5^ provide transparency in how medical PACs are aligned with this public health issue. Although it is, to our knowledge, a first in-depth assessment of this alignment and lack thereof, it is unlikely to be the last such examination in relation to firearm injury prevention goals of PACs and voices in the physician community. As greater transparency is brought to this issue by analyses such as in the article by Schuur et al,^5^ medical PACs must consider the increasing physician voice on the need to address firearm-associated morbidity and mortality in the policy arena to reduce their experience with this issue in emergency bays, operating rooms, and clinics.
